# Simplified J774A.1 macrophage assay for fungal pathogenicity demonstrates non-clinical *Nakaseomyces glabratus* strains survive better than lab strains.

**DOI:** 10.17912/micropub.biology.001266

**Published:** 2024-08-22

**Authors:** Corey M. Pezak, Christine L. Iosue, Dennis D. Wykoff

**Affiliations:** 1 Biology, Villanova University, Radnor, Pennsylvania, United States

## Abstract

*Nakaseomyces glabratus* (formerly known as *Candida glabrata* ) is the second most common cause of candidiasis, whereas the closely related yeast, *Saccharomyces cerevisiae,* causes few infections. Macrophages can control *N. glabratus* infections through phagocytosis, but in cell culture, *N. glabratus* is able to persist in macrophages better than non-pathogenic yeast. Using J774A.1 macrophages, we simplified a standard persistence/survival assay by counting yeast cells with flow cytometry and incorporating an antifungal treatment. These improvements minimized wash steps and variation so fewer replicates were needed. Here, we demonstrate that loss of *NgTUP11* does not lower pathogenicity, and that three non-clinical *N. glabratus* strains survive in macrophages better than a laboratory strain.

**Figure 1.**
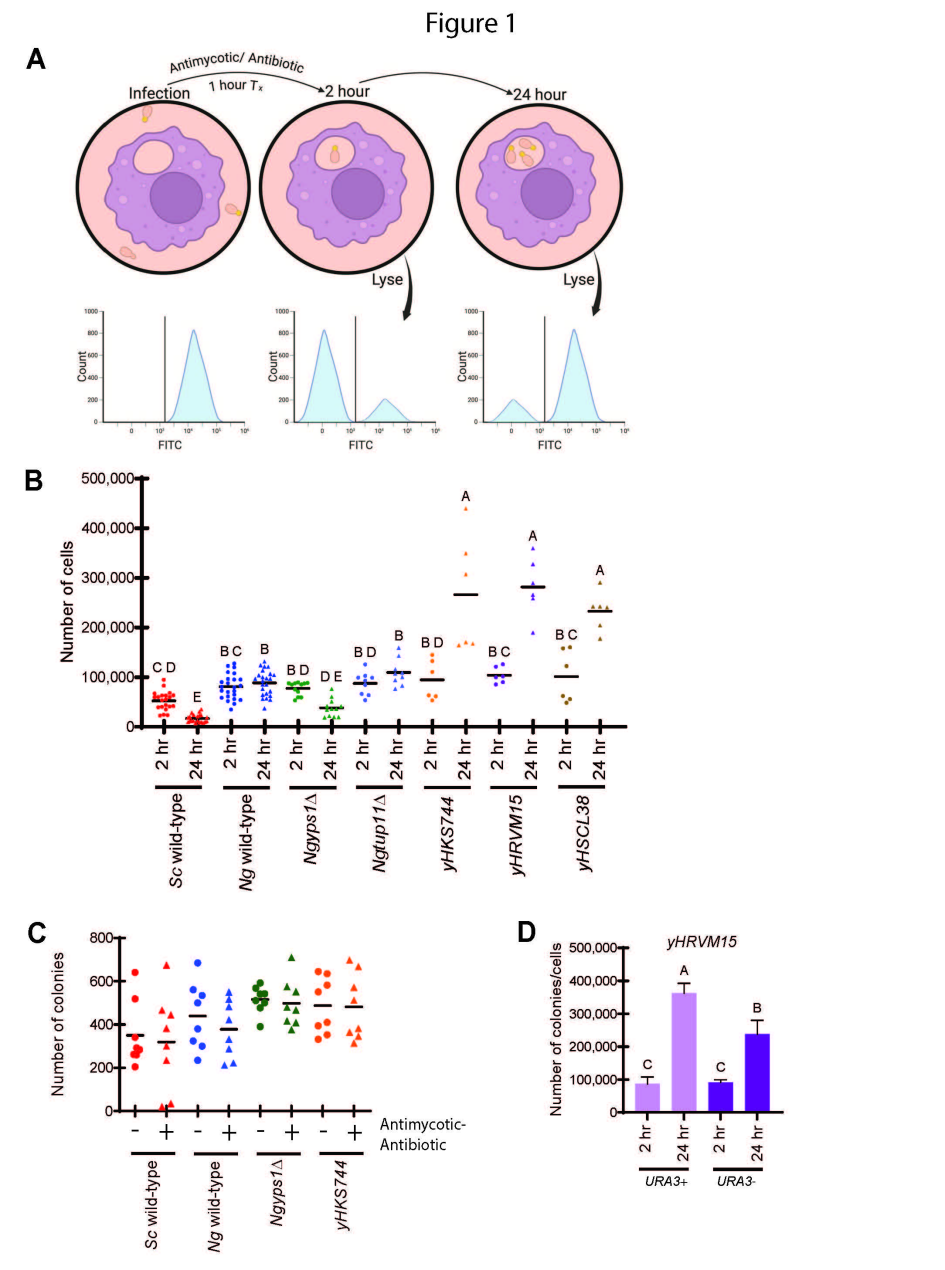
**(A)** Workflow of modified macrophage survival/persistence assay. Flow cytometry is used to obtain a cell count of fluorescent (or non-fluorescent) yeast cells, which are then used to infect macrophage cells. Antimycotic-antibiotic solution is added for half of the infection time. Macrophage cells are lysed 2 hours and 24 hours after infection and flow cytometry is used to count fluorescent yeast cells present within the lysed macrophages. The schematic was generated using BioRender. **(B)** Fluorescent yeast cells present in lysed macrophage cells were quantified by flow cytometry 2 hours and 24 hours after infection (except for *yHSCL38* which was generated by counting colony number). The number of replicates varied for each strain and replicates are represented by individual data points. A one-way ANOVA with post-hoc Tukey’s test was performed to compare all strains at both time points to yield a compact letter display to identify statistically significant differences. **(C)** Yeast strains were incubated with or without antimycotic-antibiotic for 1 hour. Each sample was plated on YEPD petri plates and colonies were counted. The data reported is two biological replicates and four technical replicates for each strain. A student’s t test was used to compare the number of colonies for each yeast strain in the presence and absence of antibiotic-antimycotic and there was no significant difference for any of the strains. **(D)** Macrophages were infected with a non-clinical *N. glabratus* strain ( *yHRVM15* ) with either the *URA3* gene intact ( *URA3* +) or replaced by integration of *NgADH1* pr-YFP ( *URA3* -). The strain without YFP was plated to determine number of colonies and the strain with YFP was counted by flow cytometry at 2 hours and 24 hours after infection. The data reported is two biological replicates and 6 technical replicates for each strain. A one-way ANOVA with post-hoc Tukey’s test was performed to compare both strains at both time points with a compact letter display.

## Description

*N. glabratus* is a commensal, opportunistic pathogen, but is phylogenetically closely related to *Saccharomyces cerevisiae* , which is not generally pathogenic (Angoulvant, Guitard, and Hennequin 2015; Gabaldón Estevan et al. 2013) *.* Many virulence factors have been identified, including adhesins and yapsins (Askari, Rasheed, and Kaur 2022; Katsipoulaki et al. 2024; Domergue et al. 2005) . Studies have used murine macrophage-like cells to assess aspects of pathogenicity and demonstrated that yapsins are required for persistence in macrophages (Kaur, Ma, and Cormack 2007; Kumar et al. 2019) . Macrophages are critical for clearing circulating fungal cells, and whether yeast cells can survive and persist in macrophages is a proxy for the ability of an organism to clear an infection.

In a standard assay, macrophages are grown in cell culture medium, yeast cells are introduced and phagocytosed, cells that are not phagocytosed are washed from the wells, macrophages are lysed after 2 hours and 24 hours, and the number of fungal cells is quantified by plating on nutrient agar (Kaur, Ma, and Cormack 2007) . Pathogenic strains persist, or even increase in cell number, in the macrophages, and less pathogenic cells are eliminated from the macrophages. While this assay sounds simple, in practice, there is variability. J774A.1 cells are not strongly adherent on tissue culture plastic, and repeated washes wash away the macrophages, removing fungal cells as well. Additionally, if a fungal cell is not phagocytosed it can rapidly outcompete the macrophages and lead to fungal overgrowth, which is readily identified by the turning of phenol red in the cell culture medium to yellow. Finally, plating and counting of fungal cells requires serial dilution, time for colonies to form, and equal plating efficiency.

To address these issues, we have incorporated two changes to the standard assay. First, the introduction of a 1-hour treatment with antimycotic (amphotericin B)-antibiotic reduced the likelihood of fungal overgrowth in the cell culture medium and removed the need for three extra wash steps in our assay. Second, by integrating into yeast cells a constitutively expressed YFP gene, we were able to immediately assess fungal census and reduce plating variability by using flow cytometry to count yeast cells ( **Figure 1A** ). We performed the persistence/survival assay with the added antimycotic-antibiotic step reducing washes. We were able to demonstrate very low variability and replicate the results published by the Cormack laboratory (Kaur, Ma, and Cormack 2007) for *S. cerevisiae* wild-type, *N. glabratus* wild-type, and *Ngyps1* Δ ( **Figure 1B** ). We present the fluorescent yeast cell count as determined by flow cytometry at 2 hours and 24 hours after infection for each strain. *S. cerevisiae* cell counts decrease after 24 hours while *N. glabratus* wild-type shows minimal change. *Ngyps1* Δ shows a decrease when compared to *N. glabratus* wild-type at 24 hours after infection, confirming that *NgYPS1* is important for survival in macrophages.

With the assay being less variable, we then queried the persistence/survival of four additional *N. glabratus* strains: *Ngtup11* Δ and three non-clinical strains isolated from various environments such as soil containing berries ( *yHSLC38* ), a sand/soil mixture ( *yHKS744* ), and a fungus ( *yHRVM15* ) (Opulente et al. 2019; Spurley et al. 2022) . We examined the *Ngtup11* Δ strain because many yapsin genes change expression in it (Bui, Iosue, and Wykoff 2022) , suggesting that there may be an increase in persistence in macrophages. Here, we demonstrate that the *Ngtup11* Δ strain persists similar to the *N. glabratus* wild-type strain, and that the three environmental strains appear to thrive in macrophages and grow better than our *N. glabratus* wild-type laboratory strain, which was isolated from a patient more than thirty years ago (Cormack and Falkow 1999) . We were unable to incorporate a YFP tag into one of the non-clinical *N. glabratus* strains, *yHSLC38* , and so we performed the assay by counting colony numbers to measure persistence. The other two non-clinical strains ( *yHKS744* and *yHRVM15* ) were assayed with the YFP tag and flow cytometry. Since the values were similar for strains with and without the YFP tag, the *yHSLC38* strain is included in **Figure 1B** .

There are many reasons that we may be observing persistence in macrophages that are not related to direct pathogenicity. One cause could be the incorporation of an antimycotic-antibiotic solution for one hour. If the solution killed the nonpathogenic appearing strains differentially, then we would observe the results in **Figure 1B** as a consequence of sensitivity to antifungal treatment. To test this, we treated *S. cerevisiae* wild-type, *N. glabratus* wild-type, *Ngyps1* Δ, and a non-clinical strain ( *yHKS744* ) with antimycotic-antibiotic for one hour and observed no significant difference in the number of colonies grown after treatment when compared to strains not exposed to the antimycotic-antibiotic ( **Figure 1C** ). Additionally, these data indicate that fungal cells are not killed with this concentration of antimycotic (25 μg/mL of Amphotericin B in Gibco antibiotic-antimycotic solution). We hypothesize that our decrease in fungal overgrowth observed with the antimycotic treatment is due to an arrest of cells in the medium, giving macrophages more time to phagocytose any remaining fungal cells in the cell culture medium, although we cannot eliminate the possibility that the antibiotics are lowering contaminations as well.

Finally, we examined whether the integration of YFP into the genome in the *URA3* locus had a significant effect on the persistence of yeast cells in macrophages. It is reasonable to think that disruption of *URA3* and the subsequent auxotrophy for uracil might impact persistence. To test this, we used an environmental strain ( *yHRVM15* ) where *URA3* is left intact or replaced with *NgADH1pr-* YFP ( **Figure 1D** ). While there is a statistically significant decrease in the number of cells after 24 hours for the strain where *URA3* is replaced with *NgADH1* pr-YFP, both strains persist in macrophages, suggesting that loss of *URA3* is not critical for persistence. Additionally, the data in **Figure 1B** (both yHKS744 and *yHRVM15* ) suggest that this replacement does not have a strong effect, as the three environmental strains appear to have similar persistence after 24 hours whether or not *URA3* is disrupted.

We believe that the incorporation of an antimycotic-antibiotic treatment for one hour during infection lowers the variability of the assay for persistence/survival in macrophage cells. With this treatment, there is no need to perform the customary wash steps, resulting in more consistent results since there is less chance of washing macrophage cells off of the cell culture substrate. We initially believed the macrophages might lower the effective concentration of antimycotic inside the macrophage cells, but **Figure 1C** demonstrates it is irrelevant. Additionally, using fluorescently labeled cells allows for accurate, rapid quantifying of yeast cells using flow cytometry. However, the incorporation of a fluorescent tag into the genome of cells is not required. We performed these assays with yeast strains containing *NgADH1pr-* YFP on a plasmid and observed similar results, suggesting that the use of plasmids may be a viable option if genomic integration is not successful. We also performed the persistence/survival assay with exogenous ketoconazole and observed similar results, but cells were more sensitive to the ketoconazole. The treatment with antimycotic-antibiotic solution is simple as it is often added to cell culture medium, and it essentially eliminates fungal overgrowth. Finally, we believe the reproducibility of the assay will allow for high-throughput analysis of strains for the ability to survive in macrophages.

## Methods

**Infection of J774.A1 Macrophage cells**

J774.A1 macrophages were cultured in T75 flasks in DMEM+Glutamax (Gibco), supplemented with 10% FBS, sodium bicarbonate, and 1x antibiotic-antimycotic (Gibco – cat #15240062 Thermo Fisher), at 37° C in a 5% CO _2_ containing incubator. Cells were passaged every 2-3 days. Prior to infection, macrophages were grown to ~90% confluency. Culture media was removed, and cells were harvested with a cell scraper and resuspended by gentle pipetting in 30 mL DMEM (supplemented as described above). 0.5 mL of this cell suspension was seeded into a 24 well cell culture plate (at a concentration ranging from 3x10 ^5^ to 5x10 ^5^ live cells/mL when counted by a DeNovix Cell Drop with the AO-PI Viability assay) and grown for ~24 hours at 37° C in 5% CO _2_ until a confluency of ~90%. For infection, medium was aspirated, and wells were washed once with DMEM + 10% FBS **without** antimycotic-antibiotic and then filled with 0.5 mL warmed DMEM + 10% FBS **without** antimycotic-antibiotic. Yeast cell numbers were quantified by flow cytometry prior to infection. Twenty microliters of yeast culture (containing 100,000 yeast cells in sterile water) was added to each well and gently mixed by pipetting. The plate was centrifuged for 1 minute at 1000 rpm and placed at 37° C in 5% CO _2_ for 1 hour. Medium was then aspirated and 0.5 mL of DMEM + 10% FBS **with** antimycotic-antibiotic was added for 1 hour at 37° C in 5% CO _2_ . Wells corresponding to 2 hours after infection were washed with DMEM + 10% FBS **without** antimycotic-antibiotic and then macrophage cells were lysed with 0.1% Triton X-100 by scraping with a pipette tip. The lysate was transferred to a 1.5 mL tube and subjected to either flow cytometry to count fluorescent yeast cells or plating onto YEPD petri plates to count colonies. DMEM + 10% FBS **without** antimycotic-antibiotic was added to wells corresponding to 24 hours after infection and the plate was incubated at 37° C in 5% CO _2_ for an additional 22 hours. Wells were washed and lysed as described and subjected to either flow cytometry or plating onto YEPD petri plates.

**Yeast strains**

To tag yeast strains with YFP, *NgADH1* pr-YFP was amplified by PCR and transformed into *N. glabratus* stains by a standard lithium acetate protocol to integrate the *NgADH1* pr-YFP into the *NgURA3* locus (Corrigan et al. 2013) . Strains defective in *URA3* were selected on solid agar medium that contained 5-FOA (1 g/L). Appropriate integration of *NgADH1* pr-YFP was confirmed by stable fluorescence and confirmatory PCR of the integration site (primers and plasmid in Reagents).

For infection of macrophage cells, all yeast cultures (strains listed in Reagents) were grown to log phase (OD _600_ between 0.2-0.8) at 30°C in YEPD medium. Cells were washed with sterile water and the cell count was obtained by flow cytometry. The yeast culture was diluted to 5,000 cells/µL in sterile water so each well of the 24 well plate could be infected with 100,000 yeast cells.

**Flow cytometry**

A flow cytometer with a 533/30 filter set (Accuri C6 Plus, BD Biosciences) was used to count fluorescent yeast cells. Yeast cells were diluted in sterile water and 50-100 µL was counted using fast fluidics. A vertical gate was used to separate fluorescent yeast cells from non-fluorescent debris found in the sample after lysis of macrophage cells. **Figure 1B and 1D** report the total cells recovered from the infection assay.

**Assaying the sensitivity of various yeast strains to the antimycotic-antibiotic**

100,000 yeast cells (in 20 µL sterile water) were incubated in 0.5 mL of DMEM + 10% FBS **with** or **without** antimycotic-antibiotic for 1 hour at 37° C in 5% CO _2_ . Each sample was plated on YEPD petri plates and colonies were counted.

## Reagents

**Table:** 

**Macrophage Cell Line**	**Genotype**	**Reference**
J774.A1	Murine macrophage cell line	Ralph and Nakoinz 1975
**Yeast Strains**	**Genotype**	**Reference**
DC10	*Scleu2* :: *ScURA3-* KANMX6- *ScADH1* pr-YFP	Iosue et al. 2016
DG234	*NgURA3* :: *NgADH1* pr-YFP	Iosue et al. 2016
DG383	*Ngyps1* ΔNATMX6	This study
DG387	*Ngyps1* ΔNATMX6, *NgURA3* :: *NgADH1* pr-YFP	This study
DG370	*Ngtup11* ΔKANMX6	Bui, Iosue, and Wykoff 2022
DG407	*Ngtup11* ΔKANMX6, *NgURA3* :: *NgADH1* pr-YFP	This study
yHSLC38	*N. glabratus* isolated from soil mixed with berries	Opulente et al. 2019
yHKS744	*N. glabratus* isolated from sand/soil sample	Opulente et al. 2019; Spurley et al. 2022
yHRVM15	*N. glabratus* isolated from a fungus	Opulente et al. 2019; Spurley et al. 2022
DG531	yHKS744, *NgURA3* :: *NgADH1* pr-YFP	This study
DG532	yHRVM15, *NgURA3* :: *NgADH1* pr-YFP	This study
**Plasmids**	**Genotype**	**Reference**
DB345	*NgADH1* pr-YFP-pRS313	Iosue et al. 2016

**Table:** 

**Primers to make strains**	**Primer number**	**Sequence**
Put *NgADH1* pr-YFP into *NgURA3* locus (amplified from *NgADH1* pr-YFP-pRS313 plasmid)	O1528	ctttgggtccatacatttgcttgcttaagactcatgttgaCGGTCCTAGTGTTCATACTG
	O1529	tcagtttcctattcttttcaagtaagcatcccagccggctcgaggcaagctaaacagatc
Check for integration into *NgURA3* locus	O479	CATCGCCAATATTAACTAACATG
	O1530	CATTGCCTCTCATATCCGTG
*Ngyps1* ΔNATMX6	O2510	ATGAAGTTTAGTTCGCTATGTATGCTGGCGTCTGTTGCTGCGGATCCCCGGGTTAATTAA
	O2511	AGATAAATGAAACCAAAAGACCAGCGACGAATAGCAAAGTGAATTCGAGCTCGTTTAAAC
Check for deletion of *NgYPS1*	O2512	GGCTCCCCTGCAAGGCTTGG
	O780	TTAATTAACCCGGGGATCCG
Put *NgADH1* pr-YFP into *NgURA3* locus (amplified from *Ngura3* :: *NgADH1* pr-YFP genomic DNA)	O2583	GCCTCATATTTACAAAGAGC
	O2584	ATTTACTAGATATTACATGC
Put *NgADH1* pr-YFP into *NgURA3* locus (amplified from *Ngura3* :: *NgADH1* pr-YFP genomic DNA)	O3883	TACACTTACAATGTCCAGTG
	O3884	TCTGAGAATGTACAATTCGG
Check for integration into *NgURA3* locus	O2520	CTAACATGTATATAAGGTTAC
	O2521	CAGTATGAACACTAGGACCG

## References

[R1] Angoulvant, A., J. Guitard, and C. Hennequin. 2015. “Old and New Pathogenic *Nakaseomyces* Species: Epidemiology, Biology, Identification, Pathogenicity and Antifungal Resistance.” *FEMS Yeast Research* , December, fov114. https://doi.org/10.1093/femsyr/fov114. 10.1093/femsyr/fov114 26691882

[R2] Askari, Fizza, Mubashshir Rasheed, and Rupinder Kaur. 2022. “The Yapsin Family of Aspartyl Proteases Regulate Glucose Homeostasis in *Candida Glabrata* .” *Journal of Biological Chemistry* 298 (2): 101593. https://doi.org/10.1016/j.jbc.2022.101593. 10.1016/j.jbc.2022.101593 PMC8844688 35051415

[R3] Bui, Lilian N., Christine L. Iosue, and Dennis D. Wykoff. 2022. “Tup1 Paralog CgTUP11 Is a Stronger Repressor of Transcription than CgTUP1 in Candida Glabrata.” *mSphere* 7 (2): e0076521. https://doi.org/10.1128/msphere.00765-21. 10.1128/msphere.00765-21 PMC9044973 35341317

[R4] Cormack, Brendan P., and Stanley Falkow. 1999. “Efficient Homologous and Illegitimate Recombination in the Opportunistic Yeast Pathogen Candida Glabrata.” *Genetics* 151 (3): 979–87. 10.1093/genetics/151.3.979 PMC1460538 10049916

[R5] Corrigan, Mary W., Christine L. Kerwin-Iosue, Alexander S. Kuczmarski, Kunj B. Amin, and Dennis D. Wykoff. 2013. “The Fate of Linear DNA in Saccharomyces Cerevisiae and Candida Glabrata: The Role of Homologous and Non-Homologous End Joining.” *PloS One* 8 (7): e69628. https://doi.org/10.1371/journal.pone.0069628. 10.1371/journal.pone.0069628 PMC3722132 23894512

[R6] Domergue, R., I. Castano, A. De Las Penas, M. Zupancic, V. Lockatell, J. R. Hebel, D. Johnson, and B. P. Cormack. 2005. “Nicotinic Acid Limitation Regulates Silencing of Candida Adhesins during UTI.” *Science* 308 (5723): 866–70. 10.1126/science.1108640 15774723

[R7] Gabaldón Estevan, Juan Antonio, Tiphaine Martin, Marina Marcet Houben, Pascal Durrens, Monique Bolotin Fukuhara, Olivier Lespinet, Sylvie Arnaise, et al. 2013. “Comparative Genomics of Emerging Pathogens in the Candida Glabrata Clade.” https://doi.org/10.1186/1471-2164-14-623. 10.1186/1471-2164-14-623 PMC3847288 24034898

[R8] Iosue, Christine L., Nicholas Attanasio, Noor F. Shaik, Erin M. Neal, Sarah G. Leone, Brian J. Cali, Michael T. Peel, Amanda M. Grannas, and Dennis D. Wykoff. 2016. “Partial Decay of Thiamine Signal Transduction Pathway Alters Growth Properties of Candida Glabrata.” Edited by Michael Polymenis. *PLOS ONE* 11 (3): e0152042. https://doi.org/10.1371/journal.pone.0152042. 10.1371/journal.pone.0152042 PMC4807840 27015653

[R9] Katsipoulaki, Myrto, Mark H. T. Stappers, Dhara Malavia-Jones, Sascha Brunke, Bernhard Hube, and Neil A. R. Gow. 2024. “Candida Albicans and Candida Glabrata: Global Priority Pathogens.” *Microbiology and Molecular Biology Reviews* 0 (0): e00021-23. https://doi.org/10.1128/mmbr.00021-23. 10.1128/mmbr.00021-23 PMC11332356 38832801

[R10] Kaur, Rupinder, Biao Ma, and Brendan P. Cormack. 2007. “A Family of Glycosylphosphatidylinositol-Linked Aspartyl Proteases Is Required for Virulence of Candida Glabrata.” *Proceedings of the National Academy of Sciences of the United States of America* 104 (18): 7628–33. https://doi.org/10.1073/pnas.0611195104. 10.1073/pnas.0611195104 PMC1863504 17456602

[R11] Kumar, Kundan, Fizza Askari, Mahima Sagar Sahu, and Rupinder Kaur. 2019. “Candida Glabrata: A Lot More Than Meets the Eye.” *Microorganisms* 7 (2): 39. https://doi.org/10.3390/microorganisms7020039. 10.3390/microorganisms7020039 PMC6407134 30704135

[R12] Opulente, Dana A., Quinn K. Langdon, Kelly V. Buh, Max A. B. Haase, Kayla Sylvester, Ryan V. Moriarty, Martin Jarzyna, Samantha L. Considine, Rachel M. Schneider, and Chris Todd Hittinger. 2019. “Pathogenic Budding Yeasts Isolated Outside of Clinical Settings.” *FEMS Yeast Research* 19 (3): foz032. https://doi.org/10.1093/femsyr/foz032. 10.1093/femsyr/foz032 PMC6702360 31076749

[R13] Ralph, P., and I. Nakoinz. 1975. “Phagocytosis and Cytolysis by a Macrophage Tumour and Its Cloned Cell Line.” *Nature* 257 (5525): 393–94. https://doi.org/10.1038/257393a0. 10.1038/257393a0 1101071

[R14] Spurley, William J., Kaitlin J. Fisher, Quinn K. Langdon, Kelly V. Buh, Martin Jarzyna, Max A. B. Haase, Kayla Sylvester, et al. 2022. “Substrate, Temperature, and Geographical Patterns among Nearly 2,000 Natural Yeast Isolates.” *Yeast (Chichester, England)* 39 (1–2): 55–68. https://doi.org/10.1002/yea.3679. 10.1002/yea.3679 PMC8881392 34741351

